# Complications of thoracoscopic TEF clipping for fistula ligation: a case report of polymer clip migration into the right main bronchus and recurrent fistula

**DOI:** 10.3389/fped.2025.1639780

**Published:** 2025-09-25

**Authors:** Annika Brandau, Jan-Hendrik Gosemann, Hannes Heublein, Ulrich Herbert Thome, Annett Bläser, Daniel Gräfe, Freerk Prenzel, Martin Lacher, Richard Wagner

**Affiliations:** ^1^ Department of Pediatric Surgery, Medical Faculty, Leipzig University, Leipzig, Germany; ^2^ Department of Anesthesiology and Intensive Care, Medical Faculty, Leipzig University, Leipzig, Germany; ^3^ Department of Neonatology, Medical Faculty, Leipzig University, Leipzig, Germany; ^4^ Department of Pediatric Radiology, Medical Faculty, Leipzig University, Leipzig, Germany; ^5^ Department of Pediatrics, Medical Faculty, Leipzig University, Leipzig, Germany

**Keywords:** esophageal atresia, tracheoesophageal fistula, thoracoscopy, clip migration, minimally invasive surgery, case report

## Abstract

**Introduction:**

Tracheoesophageal fistula (TEF) in neonates with esophageal atresia (EA) is conventionally closed by open or thoracoscopic surgery. We present a case of a rare yet potentially life-threatening complication following thoracoscopic ligation of a TEF, using a polymer clip.

**Case report:**

A term boy (GA: 42 + 1 weeks; BW: 3,110 g) underwent thoracoscopic TEF repair for Type C EA. We ligated the fistula using a polymer clip followed by primary esophageal anastomosis on the second day of life. At ten weeks of age, we readmitted the infant due to recurrent bronchitis and episodes of postprandial coughing. Flexible bronchoscopy revealed that the polymer clip, previously employed for fistula closure, had migrated into the right main bronchus and was subsequently retrieved. Despite clip removal, persistent coughing prompted further evaluation, revealing a recurrent TEF on contrast esophagography. An interdisciplinary team successfully obliterated the recurrent fistula using endoscopic chemocauterization with trichloroacetic acid (TCA) via rigid bronchoscopy. Additionally, due to a developing anastomotic stricture, the patient required four balloon dilations and a single triamcinolone injection. At the three-year follow-up, the patient is eating well and thriving normally.

**Conclusion:**

This case underscores the potential complications associated with thoracoscopic TEF closure via clipping. The application of transfixing sutures may offer a more secure and durable closure, reducing the risk of post-surgical complications such as clip migration and fistula recurrence.

## 1 Introduction

Esophageal atresia (EA) is a rare congenital malformation, classified into five subtypes according to the Gross classification. The most prevalent form, Gross Type C, characterized by EA with a distal tracheoesophageal fistula (TEF), accounts for 85%–90% of cases (1–3). In the thoracoscopic approach, TEF ligation can be performed using either a suture or a clip (metal or polymer), both of which are generally considered equivalent (4). As a result, the surgical management of TEF closure and primary esophageal anastomosis remains variable. However, experts at “ERNICA Consensus Conference on the Management of Patients with Esophageal Atresia and Tracheoesophageal Fistula” unitedly voted in favor of the transfixing suture as the preferred method for fistula ligation (5). In a systematic review TEF was ligated via suture in 38%, clipping in 30% and sutures with clipping in 30% of the cases (6). Evidence regarding complications specifically related to thoracoscopic clip ligation is limited and predominantly based on case reports, suggesting that these complications may be underreported. A previous case report described the migration of a polymer clip, used for TEF ligation, into the middle trachea, where it caused partial airway obstruction, resulting in choking and a cyanotic episode (7). Here, we present the case of a newborn with EA/TEF who also experienced translocation of a polymer clip into the right main bronchus and recurrence of the fistula following thoracoscopic TEF closure with a polymer clip, further demonstrating the potential for severe complications due to clip migration.

## 2 Case report

A male neonate, born at 42 + 1 weeks gestation with a birth weight of 3,110 g, was delivered via spontaneous vaginal delivery at a peripheral hospital by a 32-year-old healthy mother (G2P2). The Apgar scores were 9, 10, and 10 at 1, 5, and 10 min and the neonate was breathing spontaneously. Antenatal ultrasound had revealed polyhydramnios and the absence of stomach visualization, raising suspicion for EA. Postnatally, the infant presented with increased salivation and the inability to pass an orogastric tube, prompting referral to our neonatal intensive care unit.

**Figure 1:**
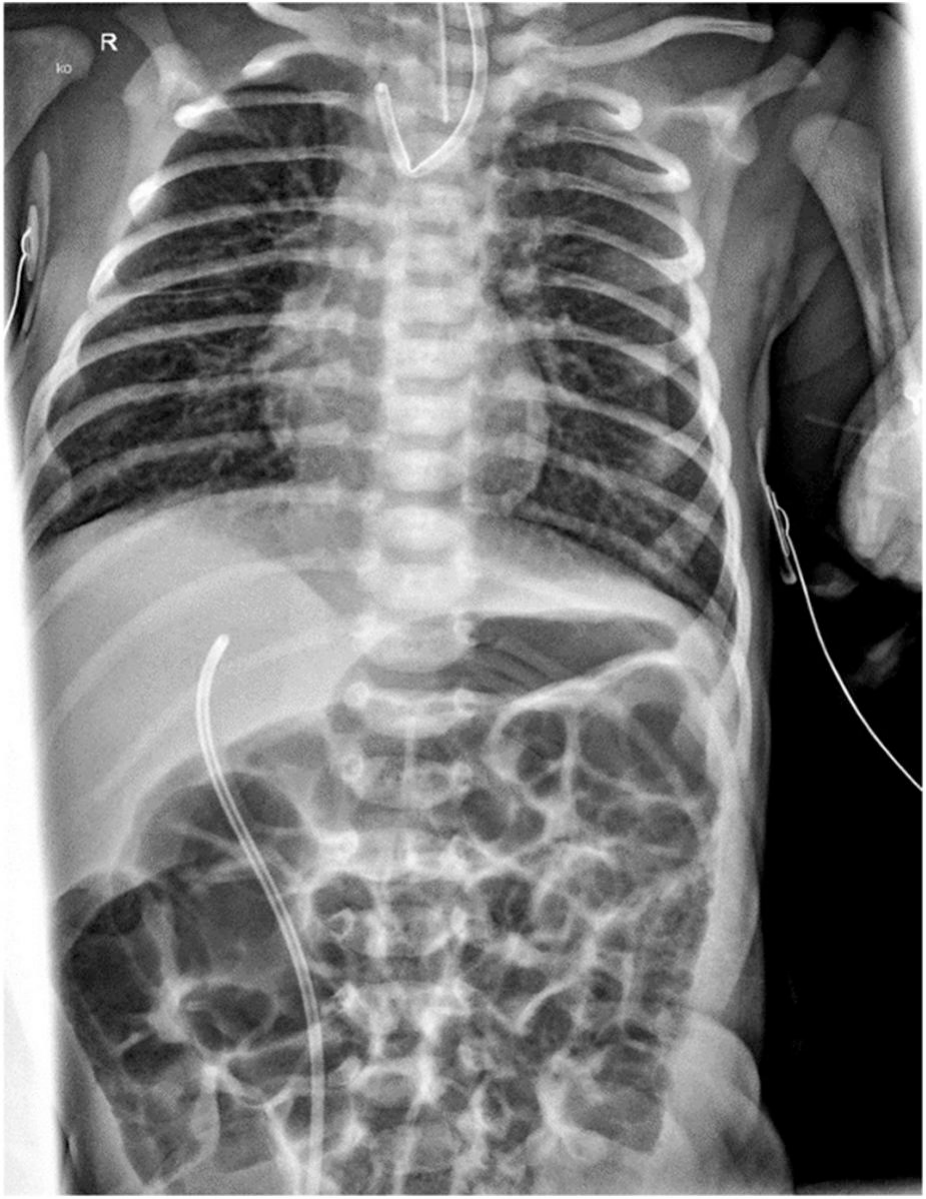
Preoperative thoracic x-ray: proximally flipped Replogl tube and normal abdominal gas distribution, indicating TEF.

A thoracic radiograph demonstrated characteristic findings of EA and distal TEF with gas in the abdomen (Figure 1). Further clinical evaluation, including sonography and echocardiography, excluded associated congenital anomalies. Subsequently, thoracoscopic ligation of the fistula with primary anastomosis was scheduled for the second day of life.

**Figure 2:**
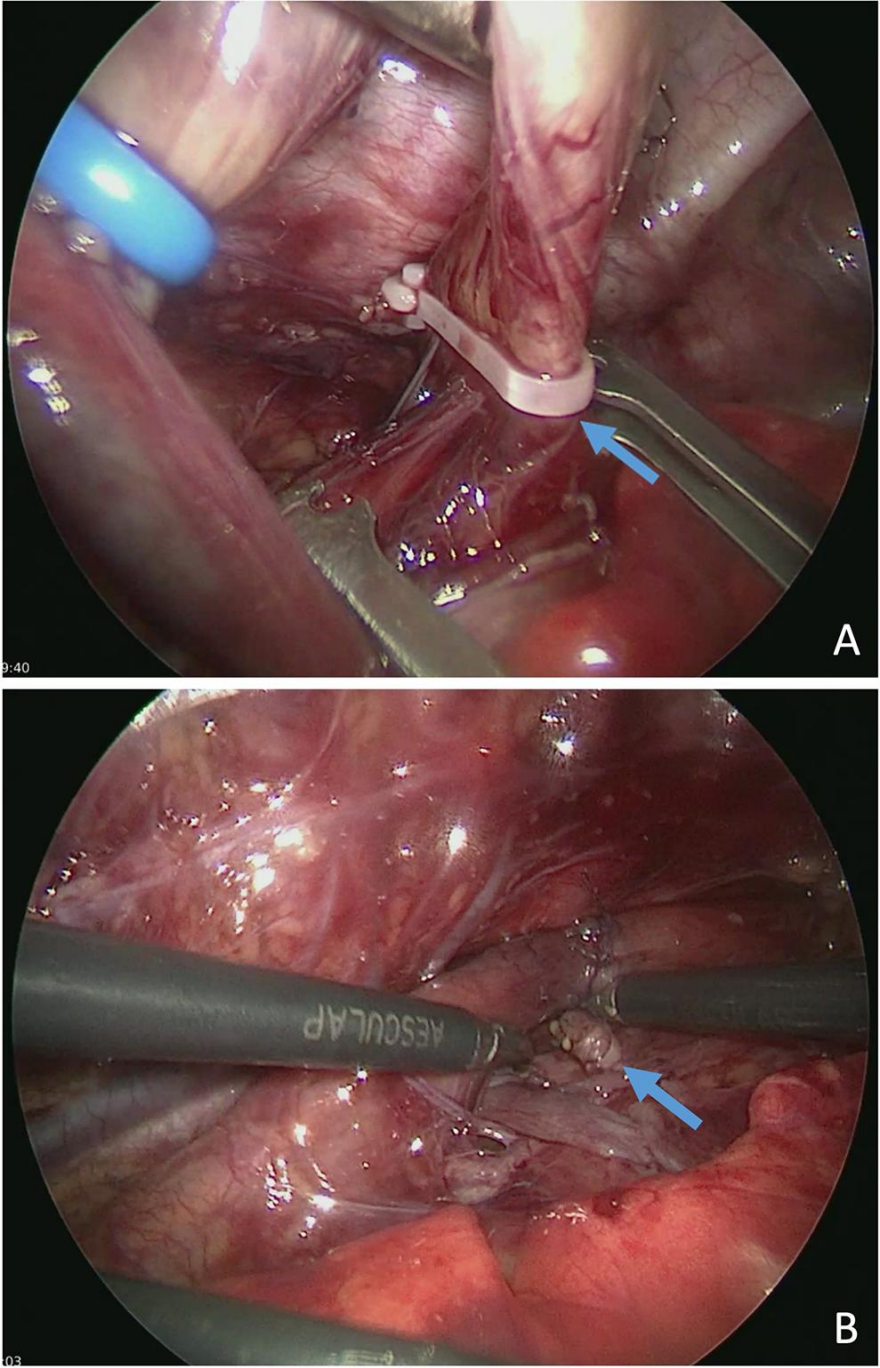
Thoracoscopic ligation of TEF using a polymer Hem-o-lok™ clip (Weck™) (arrow) before **(A)** and after primary anastomosis **(B)**.

Preoperative rigid bronchoscopy confirmed the presence of a distal TEF located just above the carina, with no evidence of a proximal fistula. The thoracoscopic procedure was performed via a standardized right-sided approach. The TEF was ligated using a 5 mm polymer Hem-o-lok™ Clip (Weck™, Teleflex™, Wayne, PA, USA), and a tension-free primary anastomosis was successfully completed using polyglactin 5.0 suture (Figure 2). A 12 Ch pleural drain was inserted in the anterior axillary line via the caudal trocar incision.

The postoperative course was uneventful. The pleural drain was removed on the second postoperative day (POD), and oral feeding was initiated gradually on the third POD, reaching full oral intake by the 10th POD. The patient was discharged in good clinical condition after two weeks.

**Figure 3:**
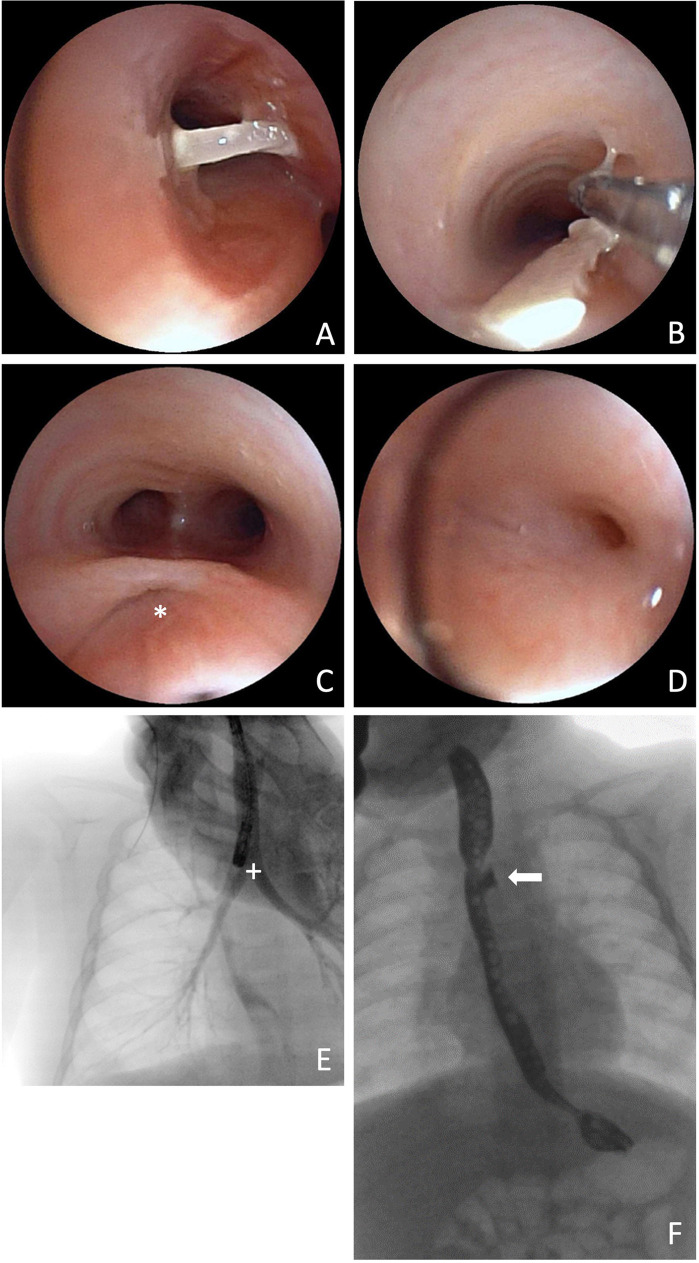
Bronchoscopic view of the clip in the right main bronchus **(A)** and its extraction via forceps **(B)**, 73 days after initial surgery. Bronchoscopic view of the fistula pouch with ostium (*) **(C)** and re-fistula ostium at the bottom of the fistula pouch **(D)**. Thoracic x-ray after administration of contrast medium directly into the fistula pouch and contrasted bronchial system (+) **(E)**. Barium swallow examination with contrasted fistula pouch (arrow) **(F)**.

However, the follow-up esophagogastroduodenoscopy, originally planned for six weeks postoperatively, was postponed due to an acute respiratory infection in order to minimize anesthesia-related risks. It was subsequently performed ten weeks postoperatively. At that time, the patient presented with recurrent episodes of bronchitis and exhibited frequent coughing episodes during breastfeeding. Physical examination revealed coarse rattling sounds that resolved after a few forceful tracheal coughs. A flexible bronchoscopy was performed, revealing migration of the polymer clip—originally used for fistula closure—into the right main bronchus. The clip was successfully retrieved using forceps (Figures 3A–B). The residual fistula tract was probed into the mediastinum but not into the esophagus. As a result, endoscopic closure was not performed during this session, and a follow-up bronchoscopy was scheduled for eight weeks later. In the bronchoscopy moderate signs of bronchitis as well as mucosal edema were observed. Cytological analysis of fluid obtained in the bronchoalveolar lavage showed evidence of increased immune cell infiltration. Subsequent esophageal endoscopy revealed an anastomotic stricture, necessitating balloon dilation to restore esophageal patency.

**Figure 4:**
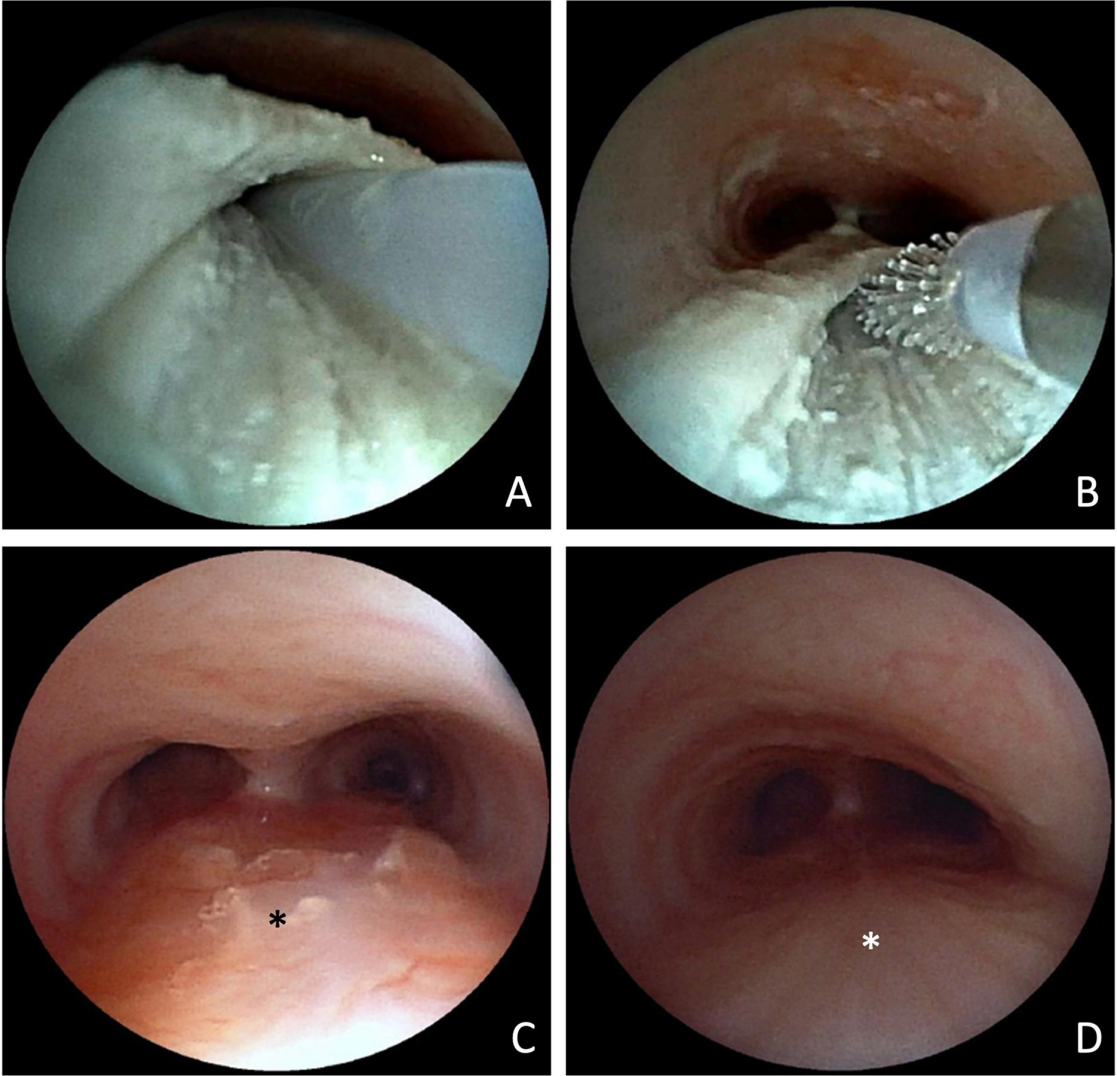
Bronchoscopic view of fistula pouch during chemocauterization with trichloroacetic acid. Application of fibrin glue **(A)** and mucosal abrasion via cytobrush **(B)**. Bronchoscopic view of the trachea with former fistula pouch (*) 14 weeks **(C)** and 15 months after the procedure **(D)**.

Two months after endoscopic removal of the migrated clip, follow-up bronchoscopy showed no evidence of a re-fistula. The former fistula pouch displayed a small ostium, which, however could not be probed, and no contrast medium passage into the mediastinum was observed even after direct application into the former fistula pouch. Despite this, the patient continued to experience food-associated coughing. At five months postoperatively, an esophageal barium swallow study was performed, revealing a recurrent TEF (Figure 3F). Subsequent bronchoscopy confirmed the diagnosis (Figures 3C–E). Endoscopic chemocauterization of the fistula was performed using a safety swab soaked in 50% trichloroacetic acid applied into the fistula pouch three times for 30s each, followed by fibrin glue application (Figure 4). Immediately the food-associated coughing resolved.

The patient required in total four balloon dilations and a single triamcinolone injection of 10 mg into the esophageal stenosis to address the anastomotic stricture. By 14 months, esophageal calibration reached 12 mm, and the child was able to feed without any further issues.

In association with the primary condition, the patient was also diagnosed with tracheomalacia, while asthma was identified as an unrelated, independent diagnosis. During follow-up, he required antibiotic therapy due to recurrent exacerbations of chronic bacterial bronchitis with evidence of bronchiectasis on MRI. However, the most recent bronchoscopy at three years of age showed no evidence of re-fistula. The fistula pouch had completely resolved, with only a fibrous scar tissue plate remaining. Under azithromycin long-term therapy, a significant improvement in bronchitic symptoms was observed. The exact origin of the bronchiectasis remains unclear and is likely multifactorial. Otherwise, at three years old, the patient has normal swallowing function and continues to develop appropriately along his growth percentile.

## 3 Discussion

EA is a congenital anomaly with an incidence of 3.16 per 10,000 live births (8), with the combination of EA and a distal TEF (Gross type C) being the most common presentation (1–3). Due to the rarity of this condition, case reports provide critical contributions to the available evidence.

Surgical intervention for EA/TEF is generally not emergent, with most procedures being electively performed within the first 48 h of life (3). Current evidence suggests that both open and thoracoscopic approaches are viable for EA/TEF repair (9, 10). However, the level of evidence supporting minimally invasive surgery (MIS) in neonates remains limited, since there are only few prospective randomized trials.

Retrospective studies suggest that MIS offers superior cosmetic and musculoskeletal outcomes compared to open repair (11). Additionally, a novel study utilizing real-time MRI indicates that, in the long-term, MIS outperforms open repair in terms of musculoskeletal complications like rib fusion and scoliosis, while patients who underwent open repair exhibit significantly smaller thoracic volume (12). These findings may underscore the growing importance of thoracoscopy, and by extension, methods for TEF closure, in the future. Thoracoscopic repair typically requires a longer operating time than open repair, so that clipping of the TEF during thoracoscopic repair may expedite the procedure, offering a simpler alternative to suturing (4). To shorten the operating time and thereby minimize the anesthesia risk, the fistula was also clipped in the case presented here.

A recent review describes that the method of TEF closure (suture, clip or combined) has no influence on the outcome (10). However, this contrasts with the expert consensus of the “ERNICA Consensus Conference” which favors the transfixing suture for TEF closure (5). This discrepancy may indicate that complications related to TEF clipping could possibly be underreported in literature, highlighting the need for further evidence.

While clipping has proven to be a safe and effective technique in various surgical contexts (13), we concur with Hagopian et al., who reported in 2014 a similar case of Hem-o-lok™ clip migration into the trachea following thoracoscopic TEF repair in a neonate with EA type C. In that case, clip migration occurred 10 weeks postoperatively, but other than in our case, without evidence of a recurrent fistula. One reason could be that Hagopian et al. placed one suture on the fistula stump since they suspected an inadequate occlusion of the fistula after clip application even though clip closure is described as sufficient (7).

A few other reports also highlighted the phenomenon of clip migration in EA/TEF. In a lecture Patkowski described a case in which a patient expectorated the clip three years after EA/TEF repair. He also noted that, in some instances, previously placed clips could no longer be visualized on follow-up chest radiographs (14). Rothenberg expresses 2012 in a publication a favorable view of fistula clipping in his retrospective review of 43 patients with EA/TEF and 3 with H-type TEF treated thoracoscopically using endoclips for TEF ligation. While no cases of clip migration or recurrent fistula were documented in that cohort, he remarked in the discussion – without citing a source – that he had heard of at least one case of clip migration into the trachea (15).

In 2019, Chiarenza et al. conducted a retrospective study evaluating the use of endoclips for TEF ligation in 32 patients with EA. In 28 patients TEF was ligated with two 5 mm titanium endoclips. They reported two cases of clip migration. The first was an intraoperative clip dislocation during mobilization of the upper esophageal pouch, which resulted in a small tracheal defect that was immediately recognized and repaired. The second was a case of late postoperative clip migration, discovered incidentally on chest x-ray one year postoperatively. The patient remained asymptomatic and did not require intervention. Based on this cohort, the incidence of late clip migration was reported as 1.7% of all applied clips (4).

In 2023, Koivusalo et al. published a retrospective study analyzing the incidence, risk factors, and outcomes of distal recurrent TEF in a cohort of 286 EA patients. Among these, only three patients were thoracoscopically treated with TEF ligation using a 5 mm metal clip. One of these three patients developed a recurrent TEF, which the authors suspected was caused by migration of the clip (16).

Another case report by Schlesinger et al. (2011) reported a case of recurrent TEF in which the previously placed clips were no longer in place on postoperative chest x-rays (17). These reports support the assumption that clip migration is a real but likely underreported complication of clip-based fistula ligation in EA/TEF repair. However, more cumulative data are needed to better quantify its incidence and assess the associated risks.

The exact mechanism of clip migration in our case remains unknown. Current evidence on its pathomechanism is largely based on laparoscopic cholecystectomy cases (7). Sheffer et al. summarized potential contributing factors such as inadequate clip closure, local inflammation - either due to a foreign body reaction or other causes - and proposed clip inversion into the clipped structure as a possible mechanism (18–21). Since in our case the clip appeared closed upon retrieval, we consider incomplete closure an unlikely cause. Hagopian et al. also hypothesized that the clip, acting as a foreign body, may have triggered an inflammatory response, potentially leading to its translocation (7). During bronchoscopic clip retrieval in the presented case, moderate signs of acute and chronic bronchitis as well as mucosal edema were observed. Additionally, analysis of bronchoalveolar lavage fluid showed a chronic and active inflammatory reaction with granulocytic and lymphocytic infiltration—findings consistent with inflammatory changes in the context of the partially recurrent fistula. At the same time, this could also support the hypothesis of an inflammatory mechanism underlying the clip migration.

Additionally, the patient had presented shortly before clip retrieval with acute obstructive bronchitis including cough and rhinorrhea, but without feeding-associated symptoms at that time. It is conceivable that increased coughing during this episode contributed to clip migration through mechanical stress. However, it cannot be completely ruled out that the infection itself may have been triggered by early stages of clip migration.

In our case the migrated clip was located in the right main bronchus. A foreign body in the tracheobronchial system is likely to cause a continuous irritation of the mucosa and may result in complications similar to those seen in foreign body aspiration. Such complications may include tracheal edema, bacterial superinfection, and pneumonia. In more severe cases, foreign body aspiration has been associated with long-term sequelae, such as bronchiectasis and bronchial strictures (22). In this case, bronchoscopy revealed findings consistent with chronic bronchitis, likely due to prolonged irritation from the migrated clip.

A structured follow-up is essential for early detection of postoperative complications. In our case, a follow-up esophagoscopy had been scheduled at 6 weeks postoperatively but was postponed due to an intercurrent infection and ultimately performed at 10 weeks, when symptoms such as feeding-associated coughing and recurrent bronchitis had emerged. As recommended by the ERNICA Consensus Conference, tracheoscopy, contrast study, and esophagoscopy were performed to assess for refistula (5). While routine bronchoscopies or contrast studies are not generally advised, they should be performed when clinical signs raise suspicion (23). In cases of suspected Re-TEF, bronchoscopy and esophagoscopy is essential to correctly asses for a potentially very small diameter fistula tract. Therefore, injection of a blue dye via bronchoscopy can help (24). To support long-term surveillance, we follow the structured aftercare schedule of the German patient support group KEKS e.V., which recommends symptom-guided assessments at 3-month intervals during the first year, and subsequent follow-ups with increasing time intervals (25). In our experience, such a framework helps ensure timely recognition of complications while diagnostic invasiveness is guided by symptoms.

Although we do not claim to have identified a novel complication, our case reinforces the potential for polymer clip migration and recurrent fistula formation following thoracoscopic TEF closure, as reported in previous studies. While clip application is considered a quick and easy technique, its convenience in the short term may come at the cost of long-term complications. This highlights the need for careful consideration when selecting a ligation method for TEF closure. While several studies report no significant difference in complication rates between TEF closure using clipping or suturing, potentially underreported adverse events—such as clip migration—may arise due to the limited number of cases studied. Case reports such as this one contribute valuable insights into rare but significant complications, informing surgical technique, postoperative care, and follow-up strategies.

Based on our experience and in agreement with Hagopian et al., transfixing sutures may be the preferable method for TEF ligation, particularly in light of the potential risks associated with polymer clip use. This perspective aligns with the recommendations of the “ERNICA Consensus Conference on the Management of Patients with Esophageal Atresia and Tracheoesophageal Fistula”, which unanimously advocated for TEF closure via transfixing suture as the preferred technique (5). The initial surgery in the case presented in our report was performed in 2021. Since then, we have adapted our technique in accordance with the ERNICA guidelines and no longer use polymer clips for fistula ligation.

## 4 Conclusions

In neonates with EA and TEF, the use of transfixing sutures for fistula closure may offer a safer alternative compared to clipping, potentially reducing the risk of rare but severe complications associated with thoracoscopic clipping of the TEF – though further evidence is needed to support a general recommendation.

## 5 Patient perspective

Up to the age of about 1 1/2 years, EA played a major role in our child's everyday life and thus also in ours. Food intake was associated with crying and there were often instances where food got “stuck”. With increasing age and after completion of the treatment of the anastomotic stricture, he likes to eat very well and usually without problems.

The recurrent infections have a greater influence, including the bronchiectasis that has now been diagnosed. The cough has been with us since his birth, with the highlight certainly being the discovery of the clip and the re-fistula. It was a great relief to learn that the treatment of the fistula was successful on the first attempt. The frequent infections, which always accompany coughing have remained. It is difficult to say to what extent he feels restricted by the cough, as often he seems not bothered by it. The side effects, such as frequent visits to the doctor, taking medication and exclusion from activities (kindergarten, playground) are certainly more significant. Permanent antibiotic therapy over the winter months has led to a significant improvement. It remains to be seen how things will continue after the therapy has been paused.

The hospitalizations were a great burden for him, his brother and us as a family. The many physical examinations, the uncertainty and also the medical procedures were new and dealing with them was not always easy. It has gotten better and there will certainly be new developments again, but he is a very funny, active child and gets along well with everything that belongs to him.

## Data Availability

The original contributions presented in the study are included in the article/Supplementary Material, further inquiries can be directed to the corresponding author.
